# Withasomniferol D, a New Anti-Adipogenic Withanolide from the Roots of Ashwagandha (*Withania somnifera*)

**DOI:** 10.3390/ph14101017

**Published:** 2021-10-02

**Authors:** Bum Soo Lee, Min Jeong Yoo, Heesun Kang, Seoung Rak Lee, Sil Kim, Jae Sik Yu, Jin-Chul Kim, Tae Su Jang, Changhyun Pang, Ki Hyun Kim

**Affiliations:** 1 School of Pharmacy, Sungkyunkwan University, Suwon 16419, Korea; kosboybs@naver.com (B.S.L.); kch970513@naver.com (M.J.Y.); hskang428@g.skku.edu (H.K.); davidseoungrak@gmail.com (S.R.L.); malin_1272@naver.com (S.K.); jsyu@bu.edu (J.S.Y.); 2 Natural Product Informatics Research Center, KIST Gangneung Institute of Natural Products, Gangneung 25451, Korea; jckim@kist.re.kr; 3 College of Medicine, Dankook University, Cheonan 31116, Korea; jangts@dankook.ac.kr; 4 School of Chemical Engineering, Sungkyunkwan University, Suwon 16419, Korea

**Keywords:** *Withania somnifera*, Solanaceae, withanolide, NMR, ECD, 3T3-L1 preadipocytes, adipogenesis

## Abstract

*Withania somnifera* (Solanaceae), well-known as ‘Indian ginseng’ or ‘Ashwagandha’, is a medicinal plant that is used in Ayurvedic practice to promote good health and longevity. As part of an ongoing investigation for bioactive natural products with novel structures, we performed a phytochemical examination of the roots of *W. somnifera* employed with liquid chromatography–mass spectrometry (LC/MS)-based analysis. The chemical analysis of the methanol extract of *W. somnifera* roots using repeated column chromatography and high-performance liquid chromatography under the guidance of an LC/MS-based analysis resulted in a new withanolide, withasomniferol D (**1**). The structure of the newly isolated compound was elucidated by spectroscopic methods, including one-dimensional (1D) and two-dimensional (2D) nuclear magnetic resonance (NMR) and high-resolution (HR) electrospray ionization (ESI) mass spectroscopy, and its absolute configuration was established by electronic circular dichroism (ECD) calculations. The anti-adipogenic activities of withasomniferol D (**1**) were evaluated using 3T3-L1 preadipocytes with Oil Red O staining and quantitative real-time polymerase chain reaction (qPCR). We found that withasomniferol D (**1**) inhibited adipogenesis and suppressed the enlargement of lipid droplets compared to the control. Additionally, the mRNA expression levels of adipocyte markers *Fabp4* and *Adipsin* decreased noticeably following treatment with 25 μM of withasomniferol D (**1**). Taken together, these findings provide experimental evidence that withasomniferol D (**1**), isolated from *W. somnifera*, exhibits anti-adipogenic activity, supporting the potential application of this compound in the treatment of obesity and related metabolic diseases.

## 1. Introduction

*Withania somnifera* (L.) Dunal (the family of Solanaceae), commonly known as ‘Ashwagandha’ or ‘Indian ginseng’, is a perennial shrub widely distributed across India, Sri Lanka, South Africa, and the Mediterranean region [1]. This plant is used in Indian Ayurveda as a traditional medicine for various inflammatory diseases, such as diabetes and nervous and reproductive system disorders [2]. Recently, it is also being consumed as a functional food to promote health and longevity by enhancing immunity against extrinsic factors, delaying aging, and strengthening the body [3,4]. In practice, the extracts of *W. somnifera* roots are consumed as a dietary supplement in several forms, including powder, liquid, tablets, and capsules. *W. somnifera* has been widely studied for therapeutic purposes and possesses several pharmacological properties, including antioxidative, analgesic, antiulcerative, antiepileptic, and antibacterial properties [5]. This plant also shows positive therapeutic activities for treating anxiety, inflammation-related diseases, cognitive and neurological disorders, hyperlipidemia, and Parkinson’s disease [6,7,8]. In addition, it possesses diverse types of chemical constituents, including alkaloids, steroids, saponins, and withanolides [9,10,11]. Taxonomically, *W. somnifera* is known to be a rich source of withanolides, which are ergostane-type steroidal lactones where the steroid skeleton is oxidized to form a δ-lactone ring in the side chain and a 2-in-1-one system in the A-ring [12]. Withanolides are reported to exhibit diverse pharmacological effects, including anti-inflammatory, antitumor, hepatoprotective, antimicrobial, and immunosuppressive effects [13,14,15,16,17,18,19], which suggests that they are the most significant constituents responsible for the medicinal properties of *W. somnifera* [20].

As part of ongoing research to discover bioactive compounds in diverse natural resources [21,22,23,24], we have investigated bioactive phytochemicals from a methanol (MeOH) extract of the roots of *W. somnifera* [25,26]. In our previous phytochemical exploration of *W. somnifera* roots, we identified six novel withanolides, namely withasilolides A–F, and seven known withanolides, and confirmed that five of the isolated withanolides showed cytotoxicity against several human cancer cells, including SK-MEL-2, HCT-15, A549, and SK-OV-3 [25]. Besides withanolides, we have also identified new phenylpropanoid esters (withaninsams A and B) in our recent study, along with phenolic compounds and alkaloids that showed anti-inflammatory potential via nitric oxide (NO) inhibition by reducing the protein expression of inducible NO synthase (iNOS) [26]. As part of ongoing research to search for bioactive natural products with novel structures from *W. somnifera*, we carried out a phytochemical exploration of *W. somnifera* roots combined with liquid chromatography–mass spectrometry (LC/MS)-based analysis. Furthermore, the chemical analysis of the methanolic extracts of *W. somnifera* roots under LC/MS-based analysis led to the isolation of a new withanolide, withasomniferol D (**1**). Herein, we describe the purification and structural elucidation of compound **1** and its effect on de novo adipogenesis and lipid metabolism in 3T3-L1 adipocytes.

## 2. Results and Discussion

### 2.1. Isolation of Compound ***1***

**Figure 1:**
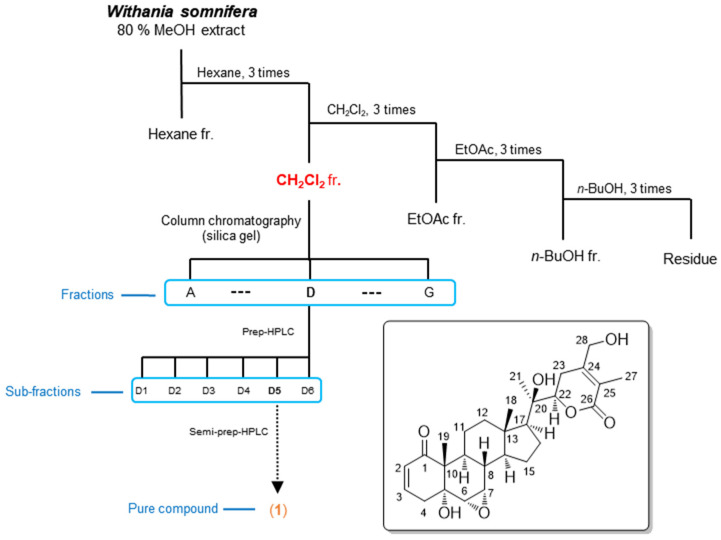
The separation scheme and chemical structure of compound **1**.

The *W. somnifera* roots were extracted with 80% aqueous MeOH under reflux to obtain the crude MeOH extract by rotary evaporation. The MeOH extract was sequentially subjected to the solvent partition procedure using four organic solvents, including hexane, dichloromethane, ethyl acetate, and *n*-butanol, to yield each solvent fraction (Figure 1). LC/MS-based analysis of the solvent-partitioned fractions obtained in combination with our in-house UV library revealed that the CH_2_Cl_2_-soluble fraction was rich in withanolides. The intensive phytochemical investigation of the CH_2_Cl_2_-soluble fraction using successive column chromatography and preparative and semi-preparative HPLC purification (Figure 1), where the isolation was monitored by LC/MS analysis, led to the isolation of a novel withanolide (**1**) (Figure 1).

### 2.2. Structural Elucidation of Compound ***1***

**Figure 2:**
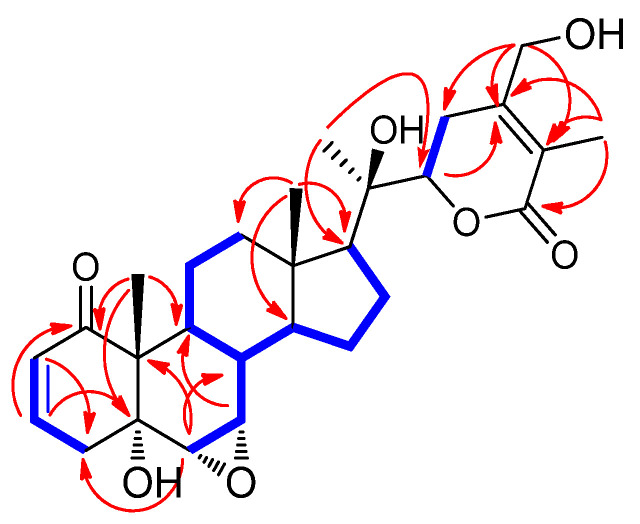
Key ^1^H-^1^H correlation spectroscopy **(**^1^H-^1^H COSY) (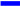) and heteronuclear multiple bond correlation (HMBC) (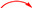) correlations for **1**.

Compound **1** was isolated as a white powder. Its molecular formula was determined to be C_28_H_38_O_7_, based on the NMR data (Table 1) and HR-ESIMS data, which showed the quasimolecular ion peak at *m*/*z* 509.2505 [M + Na]^+^ (calculated for C_28_H_38_NaO_7_, 509.2515) in the positive mode. The IR spectrum of 1 displayed distinctive absorption bands for the hydroxy (3716 cm^−1^) functional unit and *α*,*β*-unsaturated ketone (1697 cm^−1^) functional unit. The ^1^H NMR data (Table 1) of 1 combined with the data from the HSQC experiment showed the presence of proton signals for four methyls (*δ*_H_ 0.96 (3H, s), 1.18 (3H, s), 1.34 (3H, s), and 1.89 (3H, s)), seven methylenes (*δ*_H_ 1.35/2.74 (each 1H, m), 1.37/1.85 (each 1H, m, overlap), 1.37/2.08 (each 1H, m, overlap), 1.58/2.03 (each 1H, m), 2.53 (1H, dd, *J* = 19.0, 5.0 Hz)/2.68 (1H, br d, *J* = 19.0 Hz), and 4.35 (1H, d, *J* = 14.0 Hz)/4.48 (1H, d, *J* = 14.0 Hz)), and nine methines (*δ*_H_ 1.43 (1H, m), 1.54 (1H, m), 1.56 (1H, m), 1.78 (1H, m), 3.05 (1H, d, *J* = 4.0 Hz), 3.32 (1H, m), 4.22 (1H, dd, *J* = 13.5, 4.0 Hz), 5.85 (1H, dd, *J* = 10.0, 2.0 Hz), and 6.59 (1H, ddd, *J* = 10.0, 5.0, 2.0 Hz)). The ^13^C NMR data (Table 1), combined with the data obtained from the HSQC and HMBC spectra, revealed 28 carbon resonances that were classified into four methyl groups (*δ*_C_ 11.8, 13.6, 14.5, and 20.9), seven methylenes (*δ*_C_ 21.4, 21.6, 22.8, 25.7, 36.5, 40.2, and 61.2), nine methines (*δ*_C_ 34.9, 35.4, 51.8, 54.1, 55.8, 56.7, 81.8, 128.8, and 139.5), and eight non-protonated carbons (*δ*_C_ 43.7, 50.8, 73.2, 75.1, 122.2, 150.1, 166.0, and 203.3). Comprehensive scrutiny of the NMR spectral data suggested that the structure of 1 was very similar to that of withasomniferol A, previously identified in Indian ginseng by our group [25], but the apparent difference between the structures of 1 and withasomniferol A was identified in the *δ*-lactone ring due to the discrepancy in the NMR signals corresponding to C-27 and C-28. The distinctive A/B ring pattern observed in 6*α*,7*α*-epoxy-5*α*-hydroxy-1-oxowitha-2-enolide [25] was present in compound 1, similar to withasomniferol A, which was clearly determined from the NMR signals at C-1 (*δ*_C_ 203.3), C-2 (*δ*_H_ 5.85 (1H, dd, *J* = 10.0, 2.0 Hz) and *δ*_C_ 128.8), and C-3 [*δ*_H_ 6.60 (1H, ddd, *J* = 10.0, 5.0 and 2.0 Hz) and *δ*_C_ 139.5] for an *α*,*β*-unsaturated ketone; C-5 (*δ*_C_ 73.2) for a hydroxylated quaternary carbon; and C-6 (*δ*_H_ 3.05 (1H, d, *J* = 4.0 Hz) and *δ*_C_ 55.8) and C-7 (*δ*_H_ 3.32 (1H, m) and *δ*_C_ 56.7) for an epoxy functional group. The partial structure of 1 was also supported by the interpretation of the key HMBC correlations from H-2/C-3, H-3/C-1, H-3/C-5, H_3_-19/C-5, H_3_-19/C-1, and H-6/C-10, as well as the key COSY correlations from H-2 to H-4 and H-6 to H-9 (Figure 2). Importantly, the partial structure of the *δ*-lactone ring was determined by the key COSY correlations from H-22 to H-23 and the key HMBC correlations of H-22/C-24, H_3_-27/C-24, H_3_-27/C-26, H_3_-27/C-25, H_3_-28/C-23, H_3_-28/C-24, and H_3_-28/C-25 (Figure 2), which verified the *δ*-lactone ring structure of 1 (Figure 1). In conclusion, we deduced that the hydroxylation of C-27 in withasomniferol A shifted to C-28 in 1. The complete gross structure of 1 was further confirmed by a detailed inspection of the COSY and HMBC data (Figure 2).

**Figure 3:**
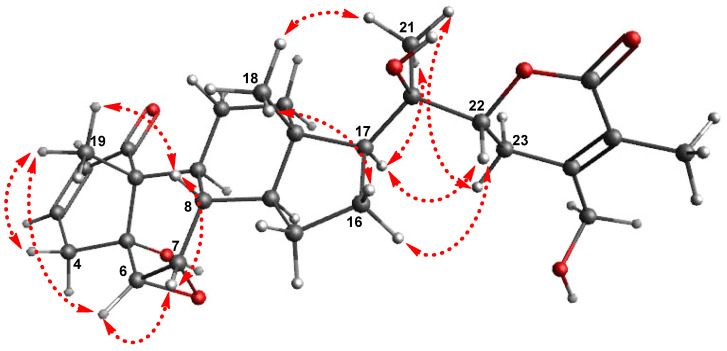
Key rotating-frame Overhauser enhancement spectroscopy (ROESY) correlations for **1**.

**Figure 4:**
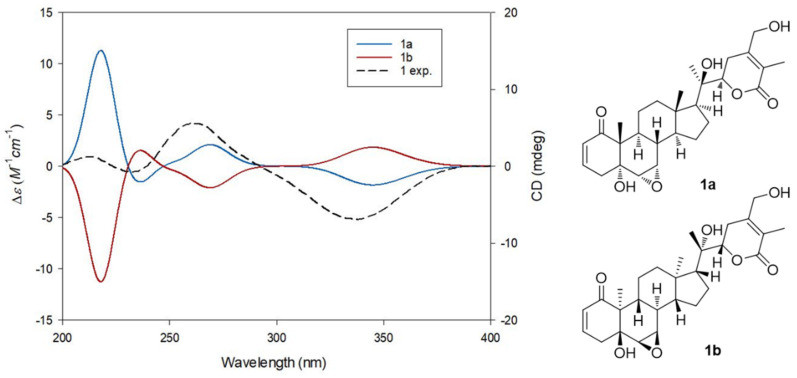
Experimental and calculated ECD data of **1**.

The absolute configuration of **1** was determined by examining the correlations obtained from the ROESY experiment, vicinal proton coupling constants in the ^1^H NMR data, and ECD data. The *α*-position of the epoxy group between C-6 and C-7 was confirmed by the ROESY correlations of H-7/H-8, H-6/H-7, H-6/ H_3_-19, and H_3_-19/H-8 and the characteristic coupling constant of 4.0 Hz between H-6 and H-7 (Figure 3), which also suggested a *trans*-linkage in the conformation of the A/B ring in **1**, based on the smaller coupling constant of 0–2 Hz for H-6, which is typical in *cis*-linkages [25,27,28]. The *trans*-linkage for the A/B ring was further verified by the negative Cotton effect around 340 nm in the ECD measurement of 1 [25], and the configurations at C-5, C-6, and C-7 were assigned accordingly, as depicted in Figure 1. The stereochemistry of H-22 (*J* = 13.5 and 3.5 Hz) was determined to be *α*-form on the basis of the typical coupling constants of H-22 reported in a previous study, where the ^1^H NMR data of H-22*α* demonstrated a doublet of doublets with two different coupling constants (*J* = 9.0–13.8 and 0.5–4.0 Hz) [25,27], while the ^1^H NMR data of H-22*β* showed two similar coupling constants (*J* = 2.5–7.0, 2.0–5.0 Hz) [28]. The *α*-orientation of H-22 was also verified by the ROESY correlations of H-16*α*/H-22 and H-17/H-22 (Figure 3). In fact, the absolute configuration of C-22 was unambiguously confirmed as *R* based on the positive Cotton effect at 260 nm derived from the n → π* transition of the *α*,*β*-unsaturated *δ*-lactone [25,28]. In addition, the ROESY correlations of H_3_-21/H-17 and H_3_-21/H-23*α* indicated the *R*-configuration of the hydroxylated quaternary carbon C-20. Finally, to confirm the absolute configuration of 1, the ECD data of two possible isomers, 1a (5*R*,6*S*,7*S*,8*S*,9*S*,10*R*,13*S*,14*S*,17*S*,20*R*,22*R*) and 1b (5*S*,6*R*,7*R*,8*R*,9*R*,10*S*,13*R*,14*R*,17*R*,20*S*,22*S*), were subjected to ECD calculations. The ECD calculation results revealed that the ECD curve of 1a (blue line) was matched with the experimentally determined ECD spectrum of 1 (Figure 4). Therefore, the chemical structure of 1, including the absolute configuration, was determined, as illustrated in Figure 1, and its trivial name was withasomniferol D.

### 2.3. Evaluation of the Anti-Adipogenic Activity of Compound ***1***

Obesity is a major health problem that results from the summation of multiple factors, including genetic, dietary, lifestyle-related, and environmental factors, which lead to the inordinate accumulation of body fat in adipose tissues [29]. Adipose tissue growth occurs with the differentiation of preadipocytes into adipocytes in the adipose tissues and the generation and collection of lipid droplets in adipocytes [30,31]. Therefore, bioactive compounds that prevent adipogenesis and lipogenesis have been considered as potential therapeutic strategies for the prevention of obesity and metabolic diseases. Recently, withaferin A, one of the most representative withanolides found in *W. somnifera*, was found to exhibit anti-adipogenic effects in 3T3-L1 adipocytes by reducing the lipid accumulation and downregulating the expression of key activators of adipogenesis, including the peroxisome proliferator-activated receptor gamma (PPARγ), CCAAT/enhancer binding protein alpha (C/EBPα), and the adipocyte fatty acid binding protein [32]. Therefore, the anti-adipogenic activities of withasomniferol D (**1**) were evaluated using 3T3-L1 preadipocytes with Oil Red O staining and qPCR.

**Figure 5:**
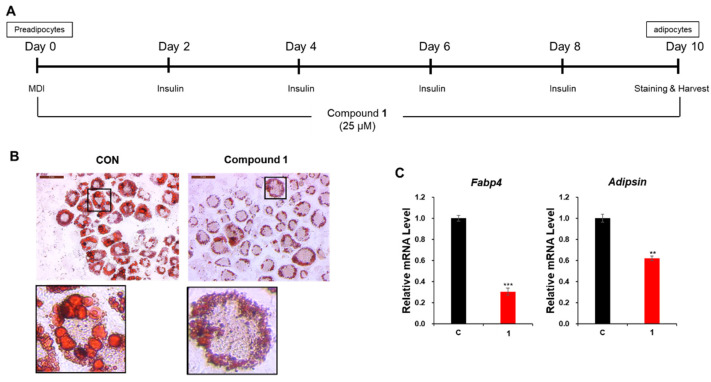
Inhibitory effects of compound **1** on adipogenesis. (**A**) Schematic representation of the differentiation of 3T3-L1 preadipocytes into adipocytes following 10 d of culture. The 3T3-L1 cells were treated with compound **1** during differentiation. (**B**) Images of adipocytes stained with Oil Red O following incubation with 25 μM of compound **1** during adipogenesis. (**C**) The relative mRNA expression levels of the fatty acid-binding protein 4 (*Fabp4*) and *Adipsin* in 3T3-L1 adipocytes incubated with 25 μM of compound **1** during adipogenesis. The data are presented as the mean ± standard error of the mean (SEM) of *n* = 3 replicates. ** *p* < 0.01, and *** *p* < 0.001.

Before assessing the effect of withasomniferol D (**1**) on adipogenesis, the 3T3-L1 preadipocytes were treated with **1** at different concentrations (0, 12.5, and 25 μM) for 24 h to evaluate their cytotoxicity. No cytotoxic effects were observed at concentrations of up to 25 μM (Figure S8). Additionally, the cytotoxicity of **1** was tested at a concentration of 25 μM for 48 h and 72 h, which also showed that compound **1** exhibited no cytotoxicity for 72 h (Figure S8). Therefore, 3T3-L1 cells were treated with **1** during adipogenesis at a concentration of 25 μM to evaluate its anti-adipogenic activity in subsequent experiments. After 10 d of differentiation, the lipid droplets within the mature adipocytes were stained using the Oil Red O working solution [33]. Microscopic examination of the stained adipocytes revealed that compound **1** significantly inhibited adipogenesis and suppressed the accumulation of lipid droplets compared to the control setup (Figure 5B). Furthermore, the mRNA expression levels of adipocyte marker genes fatty acid-binding protein 4 (*Fabp4*) and *Adipsin* were markedly reduced following treatment with 25 μM of compound **1** (Figure 5C). These results indicated that withasomniferol D (**1**) alleviated adipogenesis in 3T3-L1 preadipocytes. Interestingly, withasomniferol D (**1**) also upregulated the mRNA expression of the lipolytic genes such as hormone-sensitive lipase (*HSL*) and adipose triglyceride lipase (*ATGL*) (Figure S9). On the other hand, the mRNA expression of the lipogenic gene sterol regulatory element-binding transcription factor 1 (*SREBP1*) was downregulated following treatment with 25 μM of **1** during adipogenesis (Figure S9). These data suggest the possibility that withasomniferol D (**1**) can enhance lipid metabolism by promoting lipolysis and inhibiting lipogenesis.

## 3. Materials and Methods

### 3.1. General Experimental Procedure and Plant Material

The information for general experimental procedure and plant material is provided in Supplementary Materials.

### 3.2. Extraction and Separation/Isolation

The roots of *W. somnifera* (1.3 kg) were dried and crushed and then extracted with 80% aqueous MeOH (3.0 L × 3 d) under reflux and filtered conditions. The resultant filtrate was evaporated in vacuo using a rotavapor to generate 189 g of crude MeOH extract. The crude MeOH extract was then dissolved in 700 mL distilled water and successively solvent-partitioned with hexane, dichloromethane (CH_2_Cl_2_), ethyl acetate, and butanol to obtain 3.4, 4.5, 2.0, and 18.6 g of solvent fractions, respectively. The LC/MS analysis of each fraction was performed using a comparison of in-house-built UV spectra library, which revealed that the expected withanolides were mainly present in the CH_2_Cl_2_-soluble fraction because several peaks present in the CH_2_Cl_2_-soluble fraction showed a UV pattern (λ_max_ 200–230 nm) similar to that reported for withanolides [25] and the molecular ion peak ranging *m*/*z* 450–530. The CH_2_Cl_2_-soluble fraction (4.0 g) was subjected to silica gel chromatography column (200 g) with CH_2_Cl_2_/MeOH (50:1 → 1:1) (gradient elution solvent system) to yield seven fractions (A–G). Fraction D (160 mg) was separated by using preparative reversed-phase HPLC (Luna C18, 250 × 21.2 mm i.d., 5 μm; Phenomenex, Torrance, CA, USA) with the MeOH/H_2_O gradient system (2:3 → 1:0, flow rate: 5 mL/min) to provide six subfractions (D1–D6). Subfraction D5 (22 mg) was separated by semi-preparative reversed-phase HPLC (31% CH_3_CN) to yield compound **1** (*t_R_* 27.7 min, 1.0 mg).

Withasomniferol D (**1**)

White powder; ${\left[\alpha \right]}_{D}^{25}$+11.6 (*c* 0.05, MeOH); UV (MeOH) *λ*_max_ (log ε) 205 (3.8) nm; IR (KBr) ν_max_ 3716, 2942, 2834, 1697, 1553, 1112, and 1025 cm^−1^; ECD (MeOH) λ_max_ (∆ε) 214 (+3.6), 232 (−2.0), 260 (+16.5), and 337 (−20.3) nm; ^1^H and ^13^C NMR (800 and 200 MHz, respectively), see Table 1; positive high-resolution electrospray ionization mass spectroscopy (HR-ESIMS) *m*/*z* 509.2505 [M + Na]^+^ (calculated for C_28_H_38_NaO_7_, 509.2515).

### 3.3. Computational Analyses

The detailed procedure for the computational ECD analyses of compound **1** is included in Supplementary Materials.

### 3.4. Cell Culture and Differentiation

The information for cell culture and differentiation procedure of 3T3-L1 preadipocytes is described in Supplementary Materials.

### 3.5. Cell Viability

The 3T3-L1 preadipocytes were cultured, seeded in 6-well plates, and treated with various concentrations of compound **1** (0, 12.5, and 25 μM) for 24 h, 48 h, and 72 h. The cells were detached with a trypsin/ethylenediaminetetraacetic acid solution and diluted with phosphate-buffered saline (PBS). The number of cells was counted using the LUNA-II™ Automated Cell Counter (Logos Biosystems, Strasbourg, France).

### 3.6. Oil Red O Staining

The lipid droplets in the differentiated adipocytes were stained with the Oil Red O working solution for visualization. On day 10, the mature adipocytes were plated onto 6-well plates and fixed with 10% formaldehyde. The fixed cells were completely covered with the Oil Red O working solution to stain the lipid droplets. Then, 1 mL of PBS was added to the fixed cells before being viewed under a microscope. The lipid droplets in the stained cells were visualized and photographed using a Leica DMi1 inverted microscope (Leica Microsystems, Wetzlar, Germany). The Oil Red O stock solution was prepared by mixing 75 mg of Oil Red O powder with 25 mL of 99% isopropyl alcohol. Next, the stock solution (7.5 mL) was diluted with 5 mL of distilled water to prepare the Oil Red O working solution. The Oil Red O working solution was filtered immediately before use.

### 3.7. Quantitative Reverse Transcription-Polymerase Chain Reaction (RT-qPCR)

To extract the total RNA from mature adipocytes, we used the Easy-Blue reagent (Intron Biotechnology, Seongnam, Korea) according to the manufacturer’s instructions. Total RNA (1 μg) was reverse-transcribed into cDNA using the ImProm-II Reverse Transcription System (Promega, Madison, WI, USA). For qPCR, the generated cDNA was mixed with KAPATM SYBR^®^ FAST qPCR (Kapa Biosystems, Wilmington, MA, USA) and the primers for each gene. qPCR was performed using a CFX96 Touch^TM^ real-time PCR detector (Bio-Rad Laboratories, Hercules, CA, USA). The relative mRNA levels for each reaction were normalized to those of *β-actin*. The sequences of the used primers for qPCR are shown in Table 2.

### 3.8. Statistical Analysis

All assays were performed in triplicate and repeated at least three times. All data are presented as the standard error of the mean (SEM) for *n* = 3. Statistical significance was determined using the Student’s *t*-test (two-tailed) in Excel and was assessed on the basis of the *p*-value (* *p* < 0.05, ** *p* < 0.01, *** *p* < 0.001 vs. the control group).

## 4. Conclusions

In this study, we identified a new withanolide, withasomniferol D (**1**), present in the roots of *W. somnifera* via an LC/MS-based analysis. The structure of the new compound, withasomniferol D, was established by spectroscopic methods, including 1D and 2D NMR, HR-ESIMS, and ECD calculations. We found that withasomniferol D (**1**) effectively inhibited the differentiation of 3T3-L1 preadipocytes to adipocytes by diminishing the mRNA expression levels of *Fabp4* and *Adipsin*. Moreover, withasomniferol D (**1**) also suppressed lipid accumulation in the adipocytes. Based on these findings, we conclude that withasomniferol D (**1**) possesses the potential to prevent adipogenesis in obesity as well as obesity-related metabolic disorders.

## Figures and Tables

**Table 1:** ^1^H (800 MHz) and ^13^C NMR (200 MHz) data of compound **1** in CDCl_3_ (δ in ppm) ^a^.

Position	1	
	*δ*_H_ (*J* in Hz)	*δ*_C_
1		203.3 s
2	5.85 dd (10.0, 2.0	128.8 d
3	6.59 ddd (10.0, 5.0, 2.0)	139.5 d
4*α*	2.53 dd (19.0, 5.0)	36.5 t
4*β*	2.68 br d (19.0)	
5		73.2 s
6	3.05 d (4.0)	55.8 d
7	3.32 m	56.7 d
8	1.78 m	34.9 d
9	1.56 m	35.4 d
10		50.8 s
11*α*	2.74 m	21.4 t
11*β*	1.35 m	
12*α*	1.37 m	40.2 t
12*β*	2.08 m	
13		43.7 s
14	1.43 m	51.8 d
15*α*	1.85 m	22.8 t
15*β*	1.37 m	
16*α*	1.58 m	21.6 t
16*β*	2.03 m	
17	1.54 m	54.1 d
18	0.96 s	13.6 q
19	1.18 s	14.5 q
20		75.1 s
21	1.34 s	20.9 q
22	4.22 dd (13.5, 4.0)	81.8 d
23*α*	2.29 m	25.7 t
23*β*	2.60 m	
24		150.1 s
25		122.2 s
26		166.0 s
27	1.89 s	11.8 q
28a	4.35 d (14.0)	61.2 t
28b	4.48 d (14.0)	

^a^*J* values are in parentheses and shown in Hz; ^13^C nuclear magnetic resonance (NMR) assignments are based on the heteronuclear single quantum coherence (HSQC) and HMBC experiments.

**Table 2:** Sequences of the primers used for quantitative reverse transcription-polymerase chain reaction (RT-qPCR).

Gene	Forward Primer	Reverse Primer
*β-Actin*	5′-ACGGCCAGGTCATCACTATTG-3′	5′-TGGATGCCACAGGATTCCA-3′
*Adipsin*	5′-CATGCTCGGCCCTACATG-3′	5′-CACAGAGTCGTCATCCGTCAC-3′
*Fabp4*	5′-AAGGTGAAGAGCATCATAACCCT-3′	5′-TCACGCCTTTCATAACACATTCC-3′
*SREBP1*	5′-AACGTCACTTCCAGCTAGAC-3′	5′-CCACTAAGGTGCCTACAGAGC-3′
*ATGL*	5′-TTCACCATCCGCTTGTTGGAG-3′	5′-AGATGGTCACCCAATTTCCTC-3′
*HSL*	5′-CACAAAGGCTGCTTCTACGG-3′	5′-GGAGAGAGTCTGCAGGAACG-3′

## Data Availability

Data is contained within the article and Supplementary Materials.

## Supplementary Materials

The following are available online at https://www.mdpi.com/article/10.3390/ph14101017/s1; Figure S1: High-resolution electrospray ionization mass spectroscopy (HR-ESIMS) data of **1**; Figure S2: ^1^H nuclear magnetic resonance (NMR) spectrum of **1** (CDCl_3_, 800 MHz); Figure S3: The ^1^H-^1^H correlation spectroscopy (COSY) spectrum of **1** (CDCl_3_); Figure S4: The heteronuclear single quantum coherence (HSQC) spectrum of **1** (CDCl_3_); Figure S5: The heteronuclear multiple bond correlation (HMBC) spectrum of **1** (CDCl_3_); Figure S6: The rotating-frame Overhauser spectroscopy (ROESY) spectrum of **1** (CDCl_3_); Figure S7: The electronic circular dichroism (ECD) spectrum of **1**; Figure S8: Cytotoxicity of compound **1**; Table S1: Gibbs free energies and Boltzmann distribution of conformers **1**. General experimental procedure.
