# Supplementary material for: Withasomniferol D, a New Anti-Adipogenic Withanolide from the Roots of Ashwagandha (Withania somnifera)
Source: Pharmaceuticals (Basel). 2021 Oct 2;14(10):1017. doi: 10.3390/ph14101017 (PMC8537031; doi:10.3390/ph14101017)
Supplement: Supplementary file 1 [file pharmaceuticals-14-01017-s001.zip › pharmaceuticals-1363902-supplementary-done.pdf]

pharmaceuticals

Supplementary data

Withasomniferol D, a New Anti-adipogenic Withanolide from the Roots of Ashwagandha (*Withania somnifera*)

Bum Soo Lee [1,†], Min Jeong Yoo [1,†], Heesun Kang [1,†], Seoung Rak Lee [1], Sil Kim [1], Jae Sik Yu [1], Jin-Chul Kim [2], Tae Su Jang [3], Changhyun Pang [4,*], and Ki Hyun Kim [1,*]

[1]*School of Pharmacy, Sungkyunkwan University, Suwon 16419, Korea; kosboybs@naver.com (B.S.L.); kch970513@naver.com (M.J.Y.); hskang428@g.skku.edu (H.K.); davidseoungrak@gmail.com (S.R.L.); malin_1272@naver.com (S.K.); jsyu@bu.edu (J.S.Y.)*

[2]*KIST Gangneung Institute of Natural products, Natural Product Informatics Research Center, Gangneung 25451, Korea; jckim@kist.re.kr (J.C.K.)*

[3]*College of Medicine, Dankook University, Cheonan 31116, Korea; jangts@dankook.ac.kr (T.S.J.)*

[4]*School of Chemical Engineering, Sungkyunkwan University, Suwon 16419, Korea*

* Corresponding author: chpang@skku.edu (C.P.); khkim83@skku.edu (K.H.K.); Tel.: +82-31-290-7341 (C.P.); +82-31-290-7700 (K.H.K.)

Supporting Information Contents:

Figure S1. The HR-ESIMS data of **1**……………………………………………………………………S3
Figure S2. The ^{1}H NMR spectrum of **1** ($CDCl_3$, 800 MHz)……………………………………………………………S4
Figure S3. The ^{1}H-^{1}H COSY spectrum of **1** ($CDCl_3$)……………………………………………………………S5
Figure S4. The HSQC spectrum of **1** ($CDCl_3$)……………………………………………………………S6
Figure S5. The HMBC spectrum of **1** ($CDCl_3$)……………………………………………………………S7
Figure S6. The ROESY spectrum of **1** ($CDCl_3$)……………………………………………………………S8
Figure S7. The ECD spectrum of **1**…………………………………………………………… S9
Figure S8. Cytotoxicity of compound **1**…………………………………………………………… S10
Figure S9. The inhibitory effects of compound **1** on lipid metabolism…………………………………… S11
Table S1. Gibbs free energies and Boltzmann distribution of conformers **1** ……………………………… S12
General experimental procedure……………………………………………………………… S26

Figure S1. The HR-ESIMS data of **1**

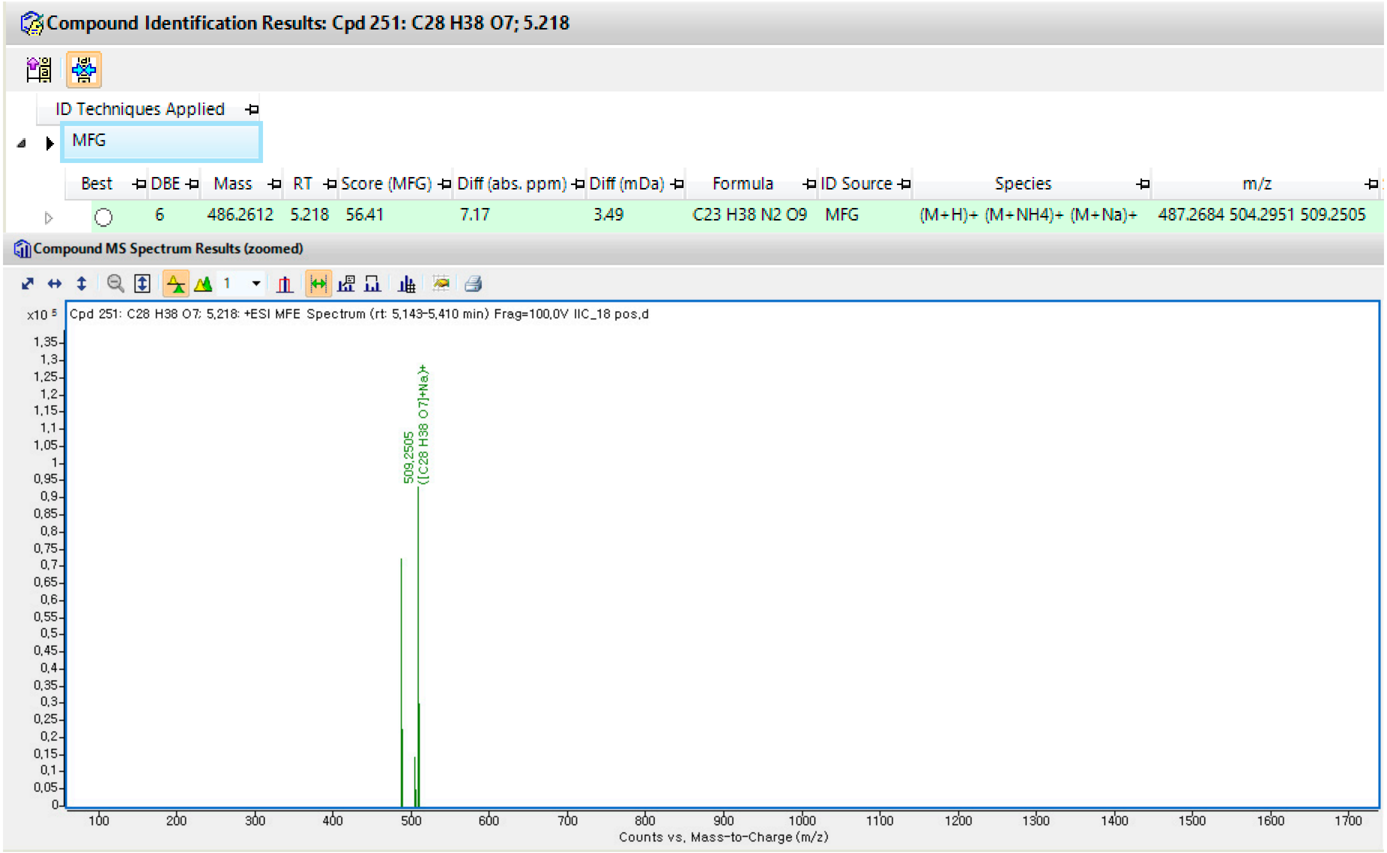

Figure S2. The ^{1}H NMR spectrum of **1** ($CDCl_3$, 800 MHz)

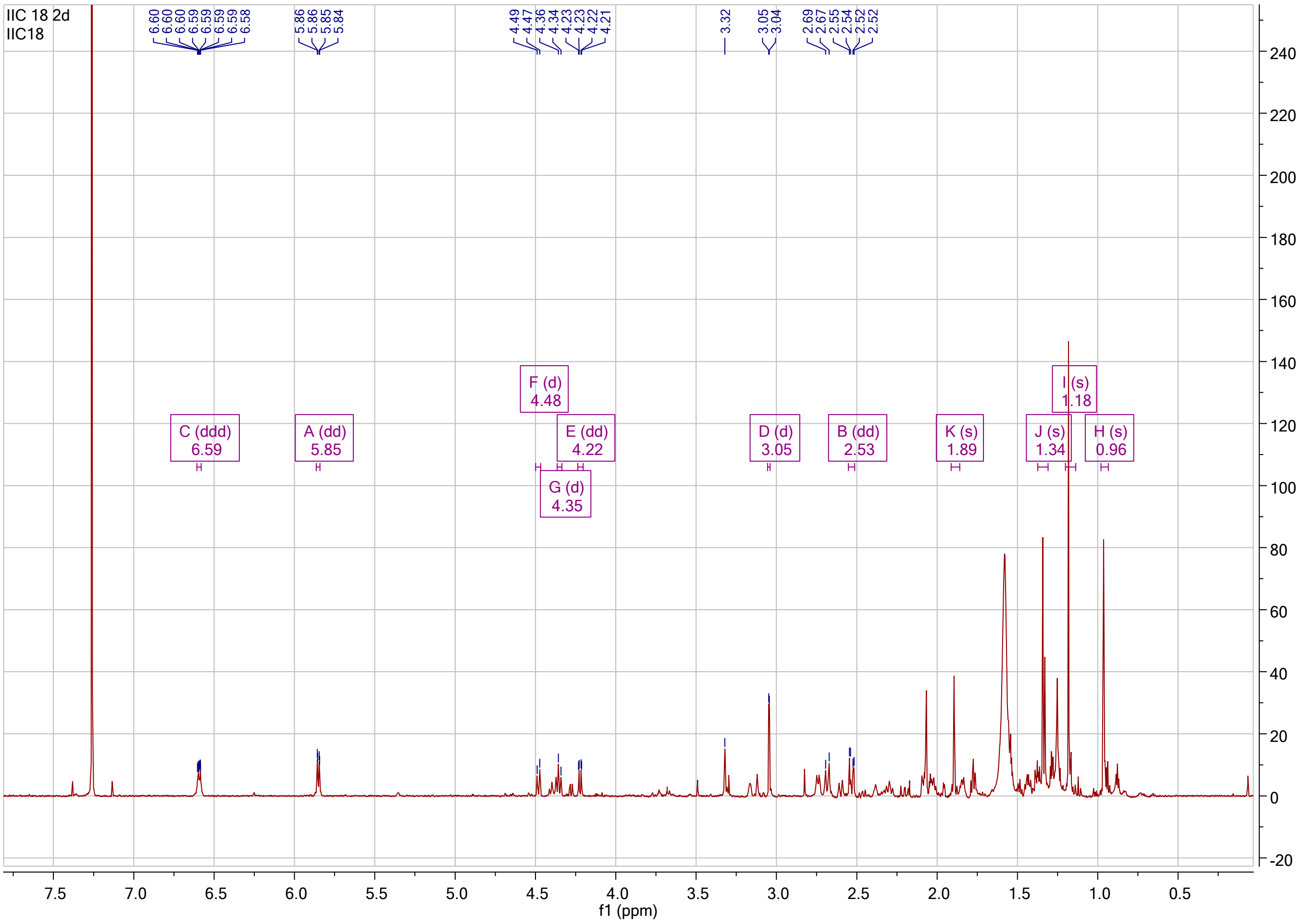

Figure S3. The ^{1}H-^{1}H COSY spectrum of **1** ($CDCl_3$)

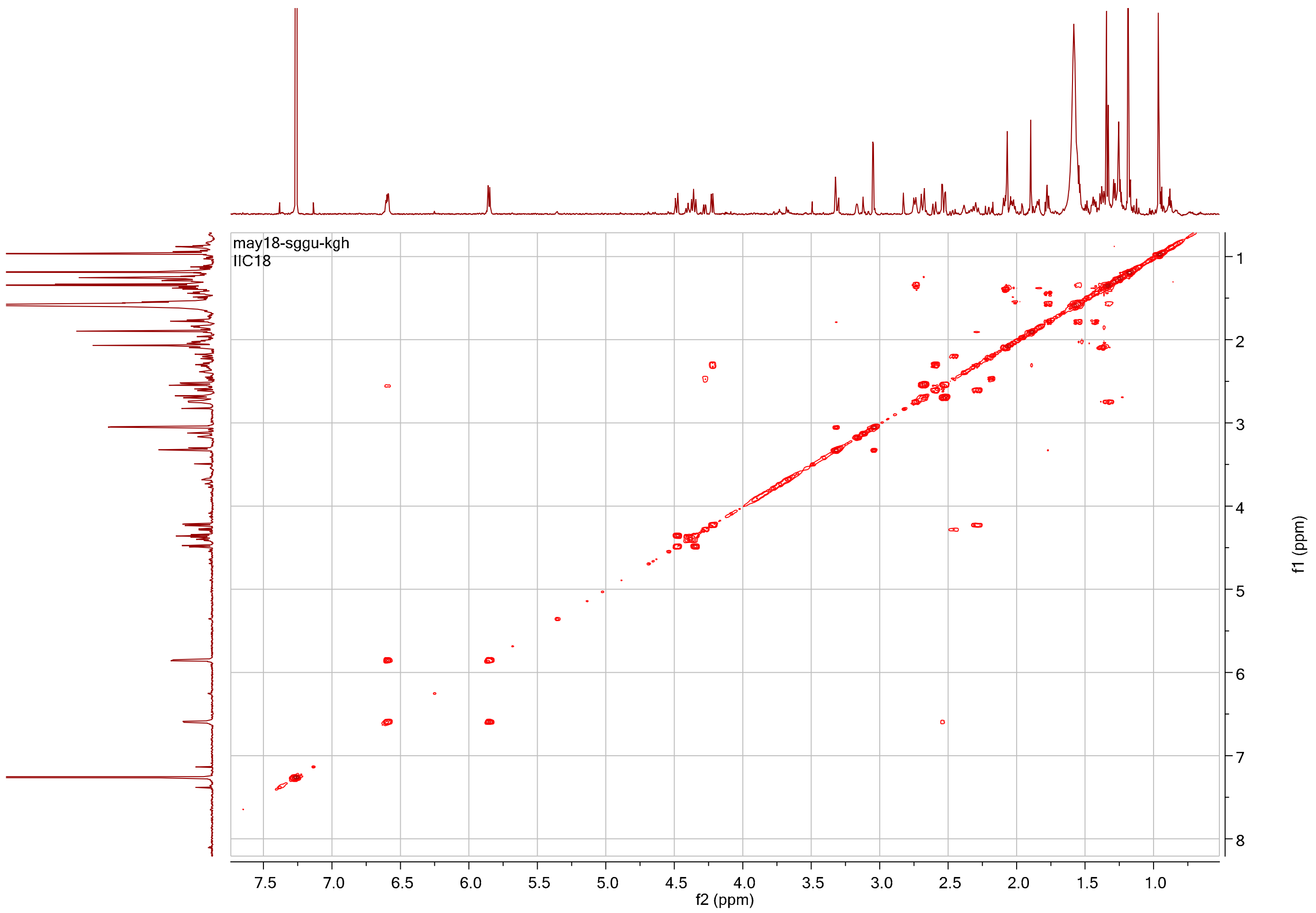
may18-sggu-kgh
IIC18
f2 (ppm)
f1 (ppm)

Figure S4. The HSQC spectrum of **1** ($CDCl_3$)

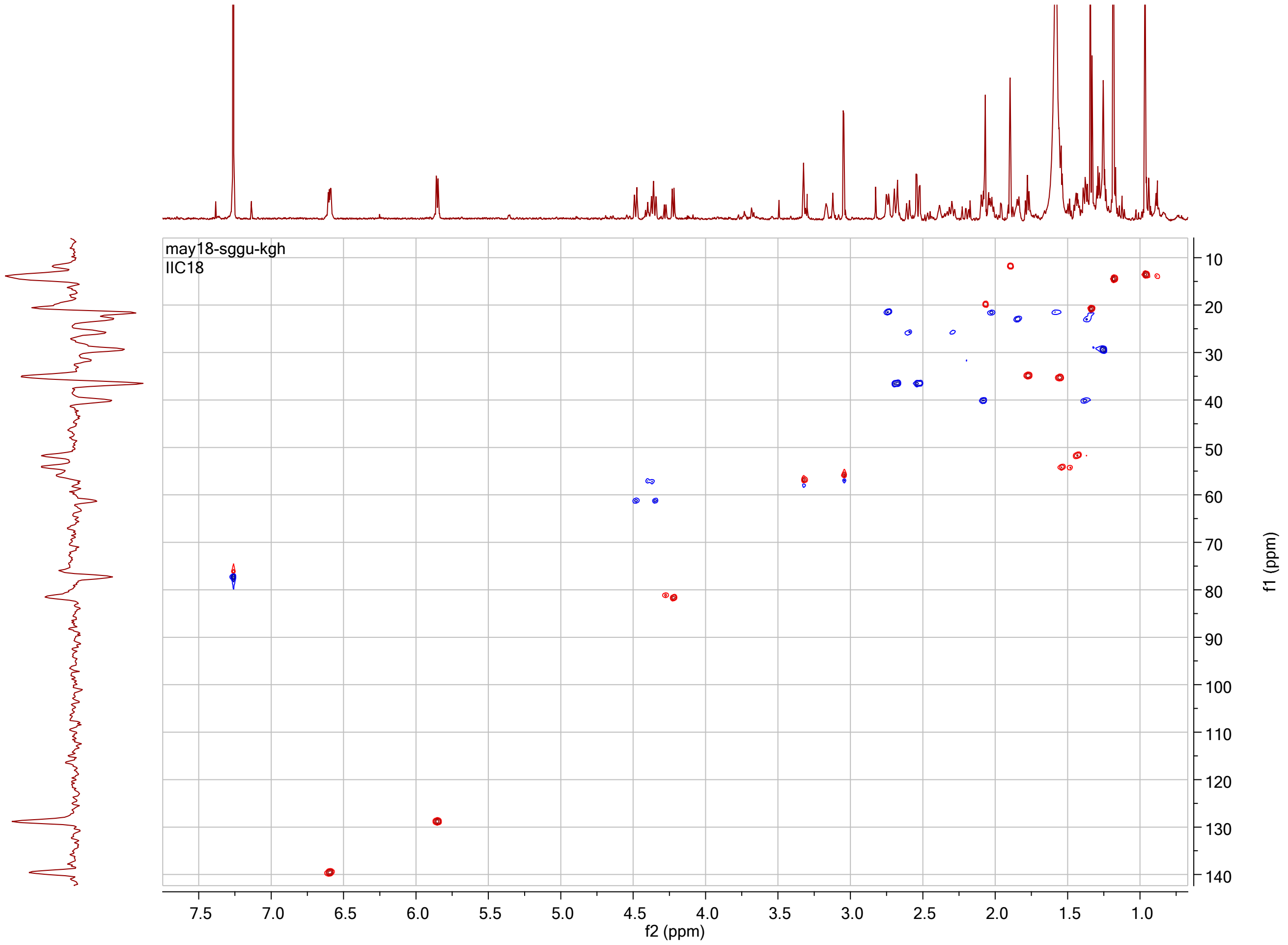

Figure S5. The HMBC spectrum of **1** ($CDCl_3$)

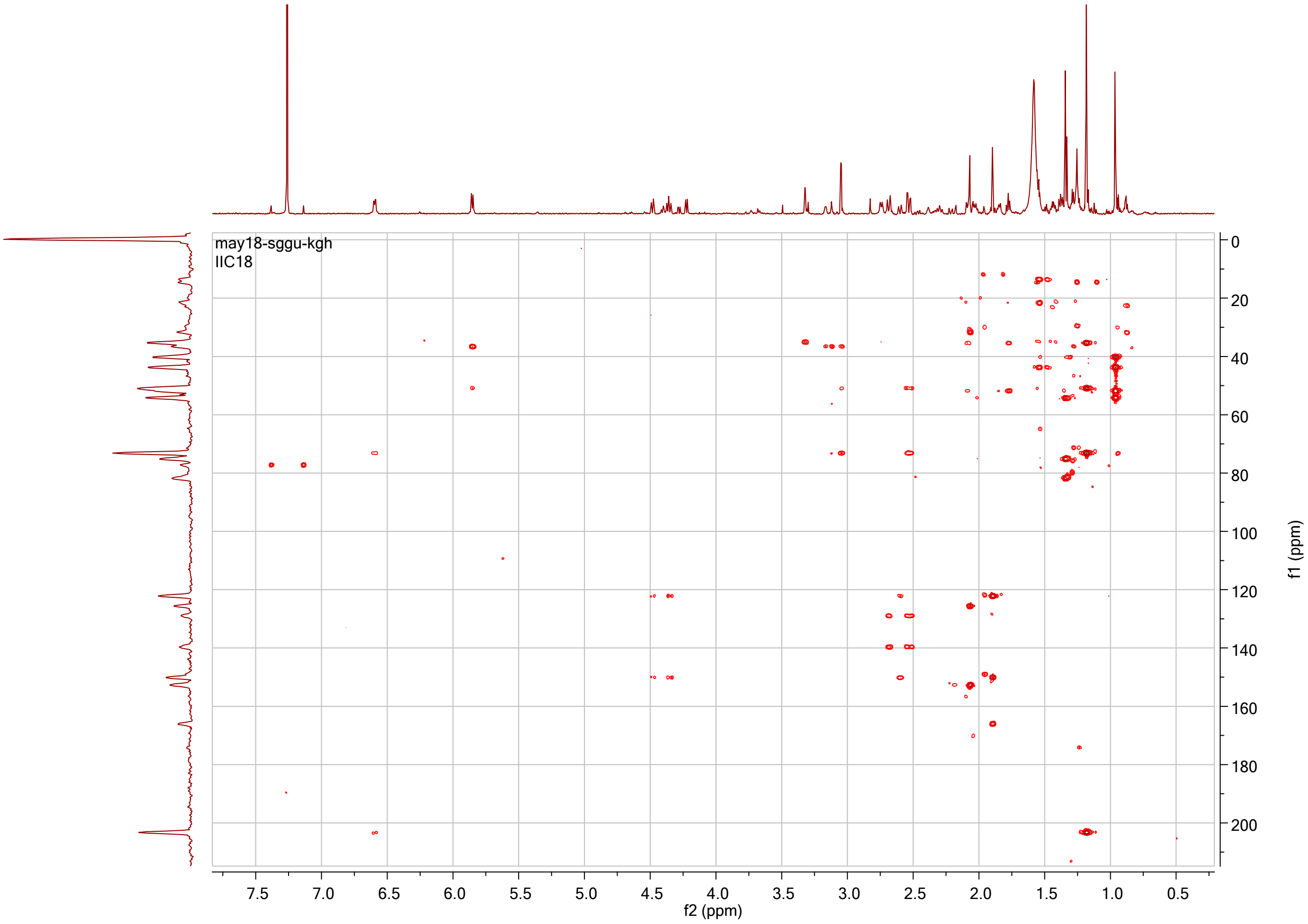

Figure S6. The ROESY spectrum of **1** ($CDCl_3$)

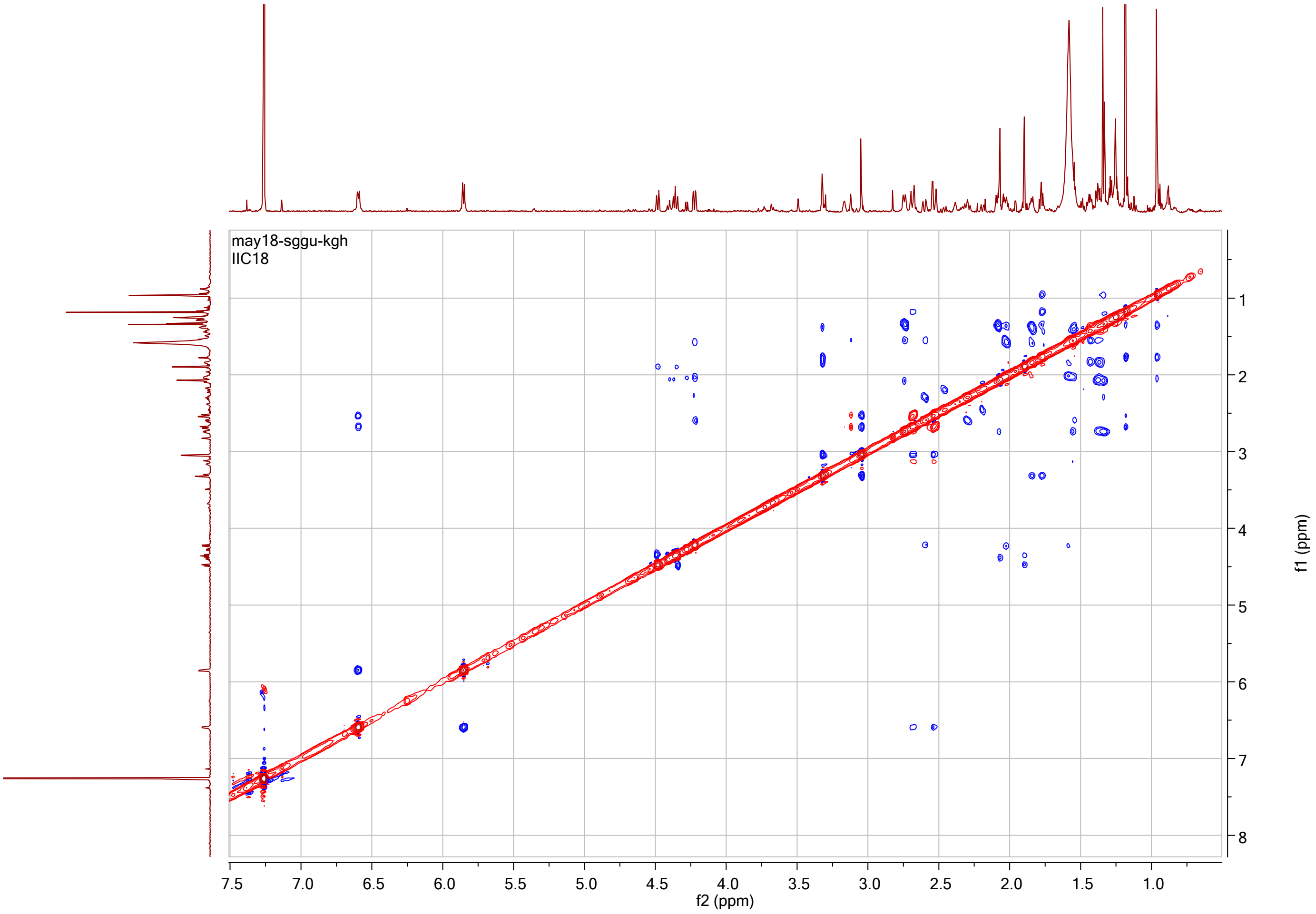

Figure S7. The ECD spectrum of **1**

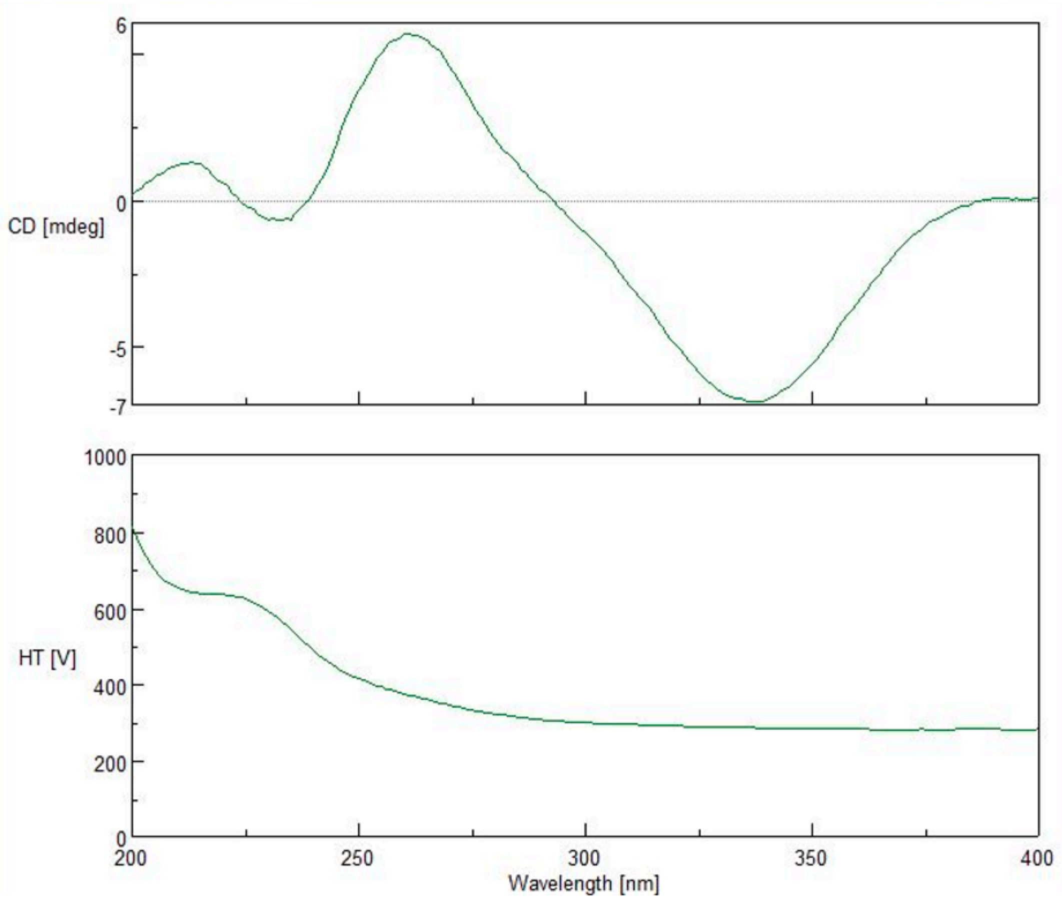

Figure S8. Cytotoxicity of compound **1**. Viability of the 3T3-L1 preadipocytes following treatment with compound **1** at concentrations of 0, 12.5, and 25 μM for 24 h, 48 h, and 72 h was measured by counting the number of cells. The data are presented as the mean ± SEM of n = 3 replicates.

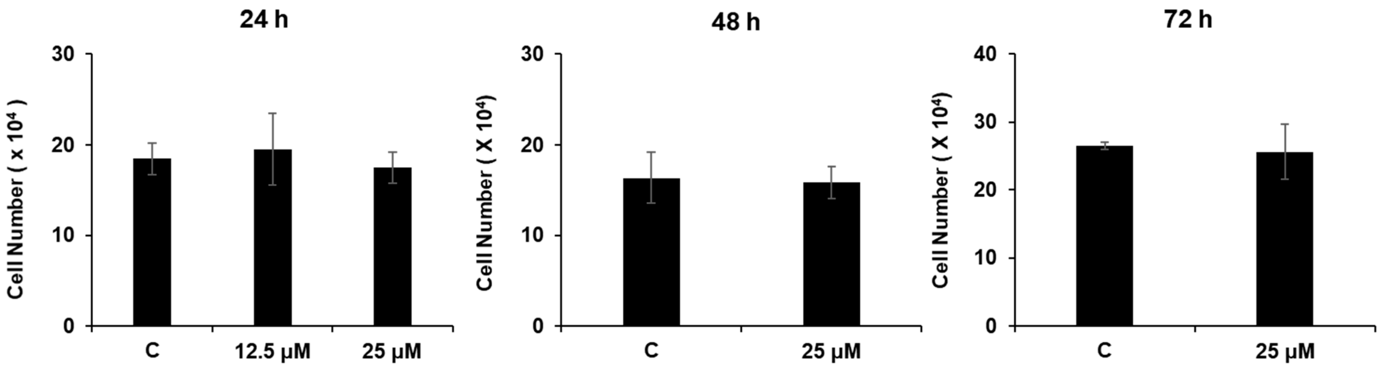

Figure S9. The inhibitory effects of compound **1** on lipid metabolism. The relative mRNA expression levels of hormone-sensitive lipase (*HSL*) (A), adipose triglyceride lipase (*ATGL*) (B), and sterol regulatory element-binding transcription factor 1 (*SREBP1*) (C) in 3T3-L1 adipocytes incubated with 25 μM of the compound **1** during adipogenesis. The data are presented as the mean ± standard error of the mean (SEM) of n = 3 replicates. **P < 0.01, and ***P < 0.001.

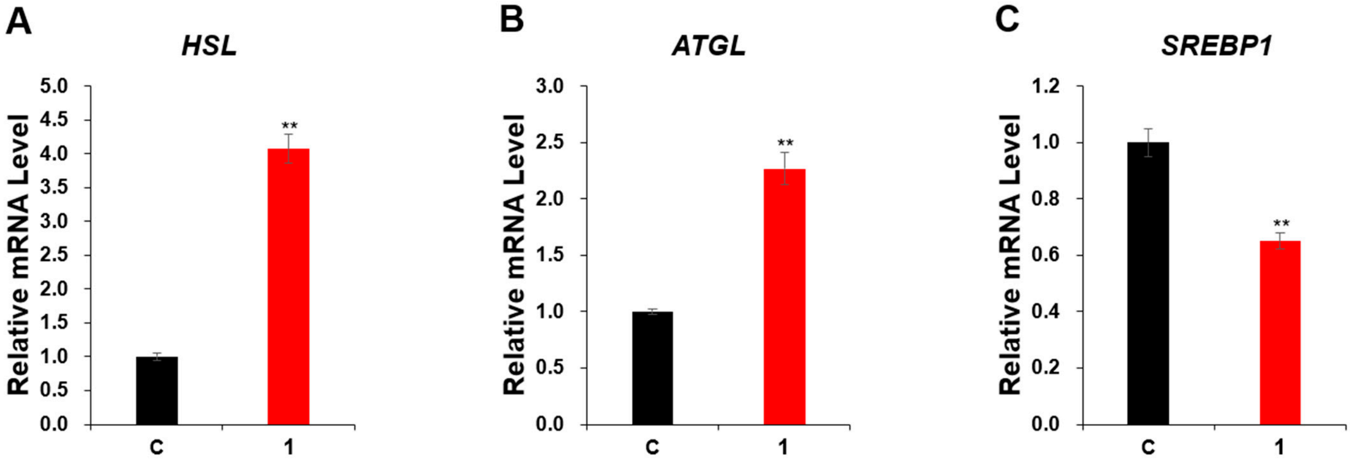

Table S1. Gibbs free energies and Boltzmann distribution of conformers **1.**

Conformers	B3LYP/6-31+G(d,p) Gibbs free energy (298.15 K)		
	G (Hartree)	∆ G (kcal/mol)	Boltzmann distribution (%)
1R-1	-1615.4131	0.00	9.64202%
1R-2	-1615.4147	-1.04	55.52672%
1R-3	-1615.4142	-0.67	30.11939%
1R-4	-1615.4124	0.42	4.71187%

Coordinates of the conformers

1_1

Coordinates (Angstroms)

X	Y	Z	atom	charge
-12.0809	0.73373	-1.33642	o	8
-10.3512	0.294866	-2.8011	c	6
-7.89475	-1.13735	-2.08465	c	6
-8.10624	-3.83011	-3.2259	c	6
-5.71652	0.435206	-3.37321	c	6
-3.07688	-0.51203	-2.61747	c	6
-2.06403	0.739046	-0.41814	o	8
-2.68179	-1.91412	-0.24956	c	6
-4.87245	-2.67843	1.414592	c	6
-5.15616	-4.6985	1.023775	h	1
-7.3771	-1.24991	0.81817	c	6
-7.13074	0.719492	1.423654	h	1
-9.4971	-2.36645	2.494462	c	6
-8.86138	-2.23716	5.342358	c	6
-6.33106	-3.53357	6.003629	c	6
-6.56465	-6.42636	5.671652	c	6
-4.28058	-2.36595	4.239859	c	6
-4.31598	-0.31586	4.595818	h	1
-1.76606	-3.32978	5.318792	c	6
-2.23049	-3.32335	8.219674	c	6
-5.11994	-2.82714	8.619974	c	6
-6.14268	-3.98475	11.11489	c	6
-5.571	-6.6268	11.11581	o	8
-9.00849	-3.61894	11.45548	c	6
-4.68258	-2.82245	13.39267	c	6
-2.66416	-3.19002	13.11468	h	1
-5.46105	-4.31258	15.56587	o	8
-4.59163	-3.66752	17.9024	c	6
-3.46419	-1.10066	18.24354	c	6
-2.25068	-0.72584	20.79425	c	6
-3.78394	0.658648	16.42229	c	6
-3.06408	3.404066	16.69054	c	6
-1.70118	4.159293	14.49211	o	8
-5.05797	-0.0335	13.95759	c	6
-4.83918	-5.15618	19.62563	o	8

-5.9364	3.013779	-2.60157	o	8
-5.99068	0.445901	-6.26623	c	6
-8.62457	1.121241	-7.08052	c	6
-10.5969	1.054496	-5.49522	c	6
-1.71126	-0.71746	-4.14916	h	1
-1.0073	-3.11105	-0.11042	h	1
-6.29626	-4.82757	-3.16093	h	1
-8.73478	-3.78651	-5.19173	h	1
-9.48088	-4.95311	-2.16843	h	1
-11.2445	-1.33625	2.146979	h	1
-9.85706	-4.33767	1.959363	h	1
-10.4339	-3.07232	6.396611	h	1
-8.76716	-0.23953	5.909963	h	1
-7.09095	-6.93784	3.739437	h	1
-8.01247	-7.18487	6.931808	h	1
-4.81511	-7.41632	6.137848	h	1
-1.34186	-5.24205	4.642465	h	1
-0.16993	-2.13056	4.777737	h	1
-1.09321	-1.86271	9.144922	h	1
-1.71053	-5.13349	9.06596	h	1
-5.40531	-0.77875	8.803174	h	1
-5.82162	-7.20854	12.83428	h	1
-9.59438	-1.65988	11.14271	h	1
-9.57369	-4.16999	13.36624	h	1
-10.0265	-4.81579	10.12157	h	1
-1.04235	-2.3402	21.24497	h	1
-3.68577	-0.64349	22.28549	h	1
-1.11644	0.993498	20.88744	h	1
-1.95347	3.725613	18.40967	h	1
-4.82655	4.506998	16.88155	h	1
-1.54409	5.976273	14.52137	h	1
-7.07719	0.443354	14.06249	h	1
-4.239	1.113988	12.44782	h	1
-4.8129	3.199903	-1.16247	h	1
-5.45154	-1.39584	-7.06587	h	1
-4.64463	1.828194	-7.02443	h	1
-8.8878	1.720218	-9.03142	h	1
-12.4791	1.640147	-6.0702	h	1

1_2

Coordinates (Angstroms)

X	Y	Z	atom	charge
-12.1689	0.496009	-1.39989	o	8
-10.4118	0.126948	-2.85089	c	6
-7.89983	-1.19284	-2.11061	c	6
-7.98761	-3.89909	-3.23731	c	6
-5.78305	0.464777	-3.39528	c	6
-3.11024	-0.36478	-2.61853	c	6
-2.16553	0.937502	-0.4186	o	8
-2.67061	-1.73884	-0.24224	c	6
-4.83693	-2.58785	1.412781	c	6
-5.03392	-4.61938	1.028785	h	1
-7.39498	-1.26683	0.795587	c	6
-7.23266	0.71444	1.390582	h	1
-9.47762	-2.45842	2.466799	c	6
-8.86268	-2.29128	5.31715	c	6
-6.28682	-3.48464	5.996358	c	6
-6.40302	-6.38512	5.671567	c	6
-4.27491	-2.24052	4.24024	c	6
-4.39499	-0.19253	4.588017	h	1
-1.72978	-3.09979	5.336199	c	6
-2.21088	-3.10428	8.234586	c	6
-5.11987	-2.72312	8.617706	c	6
-6.11175	-3.91386	11.10938	c	6
-5.43643	-6.53103	11.12076	o	8
-8.99127	-3.65695	11.43121	c	6
-4.71198	-2.69224	13.39437	c	6
-2.68018	-2.99582	13.13153	h	1
-5.45451	-4.20129	15.56703	o	8
-4.65286	-3.51375	17.91528	c	6
-3.62109	-0.9062	18.26227	c	6
-2.46891	-0.47935	20.83366	c	6
-3.9668	0.832536	16.42459	c	6
-3.25141	3.592423	16.62368	c	6
-1.64317	4.373423	14.60332	o	8
-5.18573	0.084802	13.9476	c	6
-4.87808	-5.00105	19.64226	o	8
-6.1183	3.035731	-2.64074	o	8
-6.03941	0.44684	-6.29004	c	6
-8.6948	1.00281	-7.12426	c	6
-10.6725	0.860212	-5.55067	c	6

-1.72734	-0.51804	-4.1408	h	1
-0.94775	-2.86302	-0.08784	h	1
-6.13557	-4.81612	-3.16055	h	1
-8.60899	-3.8939	-5.20586	h	1
-9.31572	-5.07594	-2.17897	h	1
-11.2622	-1.49925	2.105342	h	1
-9.75798	-4.44452	1.939488	h	1
-10.4068	-3.18282	6.367089	h	1
-8.84995	-0.28902	5.876151	h	1
-6.89567	-6.92175	3.73756	h	1
-7.82811	-7.19715	6.924056	h	1
-4.61869	-7.3038	6.15179	h	1
-1.22597	-4.99556	4.668887	h	1
-0.17996	-1.83915	4.800318	h	1
-1.1384	-1.59485	9.160496	h	1
-1.62206	-4.88902	9.089868	h	1
-5.48737	-0.68744	8.792946	h	1
-5.69899	-7.12572	12.8329	h	1
-9.64973	-1.72293	11.10734	h	1
-9.54825	-4.22279	13.33996	h	1
-9.95381	-4.89595	10.09462	h	1
-1.15042	-2.00622	21.28364	h	1
-3.92887	-0.529	22.30109	h	1
-1.4703	1.319167	20.97185	h	1
-2.42105	4.042639	18.46635	h	1
-4.96288	4.746678	16.42842	h	1
-0.05556	3.489083	14.79137	h	1
-7.2204	0.493449	14.02679	h	1
-4.38856	1.256457	12.44236	h	1
-5.01376	3.280054	-1.19578	h	1
-5.41643	-1.37432	-7.07548	h	1
-4.74973	1.881811	-7.04825	h	1
-8.97145	1.577878	-9.08051	h	1
-12.5745	1.360762	-6.14071	h	1

1_3

Coordinates (Angstroms)

X	Y	Z	atom	charge
-11.8754	1.3532	-1.2015	o	8

-10.2797	0.676068	-2.72594	c	6
-7.99307	-1.04266	-2.08046	c	6
-8.59118	-3.70686	-3.14998	c	6
-5.69354	0.218356	-3.4944	c	6
-3.16432	-1.04394	-2.82853	c	6
-1.90562	0.103844	-0.70039	o	8
-2.84504	-2.44591	-0.44896	c	6
-5.03767	-2.89629	1.323437	c	6
-5.59166	-4.87095	0.992567	h	1
-7.36403	-1.16914	0.800255	c	6
-6.8407	0.763083	1.347274	h	1
-9.53145	-1.97069	2.59085	c	6
-8.75615	-1.8733	5.405231	c	6
-6.38668	-3.47401	5.988259	c	6
-7.00383	-6.31746	5.733425	c	6
-4.28445	-2.61204	4.113145	c	6
-4.03996	-0.56851	4.423371	h	1
-1.86855	-3.87456	5.106228	c	6
-2.19874	-3.7617	8.023474	c	6
-4.97761	-2.88368	8.533926	c	6
-6.03024	-3.84974	11.09714	c	6
-5.80049	-6.54254	11.13942	o	8
-8.80805	-3.11073	11.5445	c	6
-4.3357	-2.84176	13.28561	c	6
-2.39424	-3.46896	12.92957	h	1
-5.19693	-4.1855	15.52152	o	8
-4.17286	-3.60749	17.81306	c	6
-2.71913	-1.19721	18.05173	c	6
-1.33465	-0.95186	20.52915	c	6
-2.86584	0.549704	16.19556	c	6
-1.67286	3.146666	16.28369	c	6
-3.36205	5.059234	15.42145	o	8
-4.34019	-0.01729	13.80744	c	6
-4.54851	-5.01684	19.57893	o	8
-5.5523	2.816852	-2.77385	o	8
-6.09228	0.21287	-6.37301	c	6
-8.65357	1.202598	-7.09167	c	6
-10.5462	1.414706	-5.42399	c	6
-1.90341	-1.44596	-4.41014	h	1
-1.3308	-3.84439	-0.35433	h	1

-6.91843	-4.92254	-3.13704	h	1
-9.29426	-3.61887	-5.08868	h	1
-10.0477	-4.62981	-2.01155	h	1
-11.1453	-0.72888	2.294404	h	1
-10.1668	-3.88748	2.117816	h	1
-10.3735	-2.47893	6.544975	h	1
-8.38047	0.104882	5.921081	h	1
-7.69215	-6.78669	3.841655	h	1
-8.46711	-6.86463	7.081775	h	1
-5.37262	-7.51736	6.131377	h	1
-1.72354	-5.83645	4.45506	h	1
-0.1568	-2.90158	4.47209	h	1
-0.83882	-2.44906	8.867633	h	1
-1.88169	-5.61061	8.886899	h	1
-4.98709	-0.81203	8.676606	h	1
-6.05752	-7.0575	12.87808	h	1
-9.15383	-1.0996	11.20596	h	1
-9.35541	-3.54773	13.48966	h	1
-10.0258	-4.19405	10.2834	h	1
0.100438	-2.43497	20.69954	h	1
-2.64148	-1.23247	22.10627	h	1
-0.39439	0.869553	20.74423	h	1
-0.07363	3.203605	14.9631	h	1
-0.9272	3.593583	18.16394	h	1
-4.73116	5.174333	16.62534	h	1
-6.27657	0.70292	13.98321	h	1
-3.49718	1.005107	12.21918	h	1
-4.35439	2.88486	-1.38562	h	1
-5.8262	-1.69642	-7.15211	h	1
-4.61755	1.399065	-7.21873	h	1
-8.92566	1.795234	-9.04323	h	1
-12.3634	2.223685	-5.93317	h	1

1_4

Coordinates (Angstroms)

X	Y	Z	atom	charge
-11.9012	1.281765	-1.20229	o	8

-10.2802	0.642903	-2.7164	c	6
-7.96871	-1.03881	-2.06175	c	6
-8.5057	-3.70447	-3.1596	c	6
-5.67949	0.277084	-3.44206	c	6
-3.13281	-0.94086	-2.75971	c	6
-1.91922	0.214233	-0.60945	o	8
-2.81082	-2.35578	-0.38841	c	6
-5.01306	-2.86549	1.355638	c	6
-5.52573	-4.84653	0.997911	h	1
-7.36662	-1.17789	0.824223	c	6
-6.88608	0.758915	1.39383	h	1
-9.53714	-2.03741	2.584556	c	6
-8.79283	-1.95454	5.407768	c	6
-6.4009	-3.51784	5.999746	c	6
-6.96283	-6.3694	5.708786	c	6
-4.29533	-2.59817	4.156198	c	6
-4.09083	-0.55405	4.49098	h	1
-1.86824	-3.829	5.161368	c	6
-2.23062	-3.75139	8.075868	c	6
-5.03018	-2.92903	8.566452	c	6
-6.09051	-3.94321	11.10775	c	6
-5.81206	-6.63226	11.12115	o	8
-8.88552	-3.26045	11.5363	c	6
-4.43666	-2.92996	13.32455	c	6
-2.48005	-3.51591	12.9792	h	1
-5.29017	-4.32033	15.53304	o	8
-4.2718	-3.76047	17.8335	c	6
-2.9046	-1.31306	18.11888	c	6
-1.56786	-1.03423	20.61997	c	6
-3.10076	0.462511	16.29733	c	6
-1.96421	3.075536	16.49075	c	6
-3.57296	4.796571	15.19366	o	8
-4.49699	-0.11034	13.86968	c	6
-4.592	-5.21958	19.57019	o	8
-5.59514	2.872124	-2.69979	o	8
-6.04883	0.28828	-6.32451	c	6
-8.62166	1.233544	-7.06177	c	6
-10.5346	1.396132	-5.41184	c	6
-1.84789	-1.30472	-4.33129	h	1
-1.26978	-3.72433	-0.28892	h	1
-6.81031	-4.88806	-3.14119	h	1
-9.19264	-3.61312	-5.10396	h	1

-9.9545	-4.66463	-2.04241	h	1
-11.1705	-0.82257	2.283423	h	1
-10.1317	-3.96068	2.085775	h	1
-10.4101	-2.60076	6.524862	h	1
-8.45838	0.024686	5.947319	h	1
-7.61573	-6.83307	3.80308	h	1
-8.43484	-6.95547	7.031115	h	1
-5.31613	-7.5435	6.119667	h	1
-1.68335	-5.78142	4.491951	h	1
-0.16678	-2.82089	4.554405	h	1
-0.90359	-2.42314	8.947608	h	1
-1.88928	-5.6027	8.924506	h	1
-5.0792	-0.8597	8.732338	h	1
-6.0638	-7.16793	12.8545	h	1
-9.26459	-1.25198	11.21977	h	1
-9.44184	-3.72969	13.47125	h	1
-10.0716	-4.35142	10.25154	h	1
-0.31241	-2.64624	20.94112	h	1
-2.93094	-1.0463	22.1778	h	1
-0.44206	0.689187	20.73379	h	1
-0.06531	3.033564	15.6211	h	1
-1.71874	3.630919	18.47393	h	1
-2.74909	6.422887	15.15586	h	1
-6.43971	0.59936	14.01207	h	1
-3.62923	0.935665	12.31177	h	1
-4.41584	2.949894	-1.2962	h	1
-5.7372	-1.60888	-7.11629	h	1
-4.589	1.510108	-7.14511	h	1
-8.88585	1.834879	-9.01177	h	1
-12.3615	2.175164	-5.93293	h	1

General experimental procedure

Optical rotations were measured using a JASCO P-1020 polarimeter (JASCO Corporation, Hachioji, Tokyo, Japan). Ultraviolet (UV) spectra were measured using an Agilent 8453 UV-visible spectrophotometer (Agilent Technologies, Santa Clara, CA, USA). Infrared spectra were recorded using a Bruker IFS-66/S Fourier-transform infrared spectrometer (Bruker, Billerica, MA, USA). The experimental electronic circular dichroism (ECD) spectra were measured using a quartz cuvette with an optical path length of 1 mm and a JASCO J-1500 spectropolarimeter (JASCO Corporation). For the nuclear magnetic resonance (NMR) studies, including ^{1}H-^{1}H correlation spectroscopy (^{1}H-^{1}H COSY), heteronuclear single quantum coherence (HSQC), heteronuclear multiple bond correlation (HMBC), and rotating-frame Overhauser spectroscopy (ROESY) experiments, the spectra were determined using a Varian UNITY INOVA 800 NMR spectrometer operating at 800 MHz (^{1}H) and 200 MHz (^{13}C) with chemical shifts given in ppm (δ). Preparative high-performance liquid chromatography (HPLC) was performed using a Waters 1525 Binary HPLC pump with a Waters 996 photodiode array detector (Waters Corporation, Milford, MA, USA) and an Agilent Eclipse C_{18} column (250 × 21.2 mm, 5 µm; flow rate: 5 mL/min; Agilent Technologies). Semi-preparative HPLC was performed using a Shimadzu Prominence HPLC System with SPD-20A/20AV Series Prominence HPLC UV-Vis detectors (Shimadzu, Tokyo, Japan) and a Phenomenex Luna C18 column (250 × 10 mm, 5 µm; flow rate: 2 mL/min; Phenomenex, Torrance, CA, USA). LC/MS analysis was performed on an Agilent 1200 Series HPLC system equipped with a diode array detector and 6130 Series electrospray ionization (ESI) mass spectrometer (Agilent Technologies) using an analytical Kinetex C18 100 Å column (100 × 2.1 mm, 5 µm; flow rate: 0.3 mL/min; Phenomenex). Silica gel 60 (230−400 mesh; Merck, Darmstadt, Germany) and RP-C_{18} silica gel (230−400 mesh; Merck) were used for column chromatography. Precoated F254 silica gel plates and RP-18 F254s plates (Merck) were used for thin-layer chromatography (TLC). The spots on the TLC plate were detected under UV light or by heating after spraying with anisaldehyde-sulfuric acid.

Plant material

The roots of *W. somnifera* were acquired from Seong-geo-san Farm, Cheonan, South Korea, in October 2016, and the natural source was authenticated by one of the authors (K.H.K.). A voucher specimen of the material (IDG-2016) was placed in the herbarium of the School of Pharmacy, Sungkyunkwan University, Suwon, South Korea.

Computational analyses

All conformers reported in this study were generated using the MacroModel module (version 2019–3; Schrödinger LLC) with mixed torsional/low-mode sampling and the MMFF94 force field. All searches were initially performed in the gas phase. The window limit was set to 10 kJ/mol energy, and the maximum number of steps was set to 10,000 to thoroughly explore all the potential conformers. The Polak–Ribière conjugate gradient protocol was established with 10,000 maximum iterations and a convergence threshold of 0.001 kJ (mol Å)$^{-1}$

on the rms gradient to minimize the conformers [24]. The conformers reported in this study (within 50 kJ/mol in the MMFF force field) were selected for geometry optimization by Tmolex 4.3.1, using DFT settings of B3-LYP/6-31+G(d,p). The ECD calculations for conformers **1a** and **1b** were performed at identical theoretical levels and basis sets. The calculated ECD spectra were simulated by superimposing each transition, where σ is the width of the band at a height of 1/e, and ΔE_i and R_i are the excitation energies and rotatory strengths, respectively, for transition *i*. In the present study, the value of σ was 0.10 eV for conformers **1**. The calculated excitation energies and rotational strengths for the ECD spectra were calculated based on the Boltzmann populations of conformers, and the ECD was visualized using SigmaPlot, version 14.0.

$$\Delta\epsilon(E) = \frac{1}{2.297 \times 10^{-39}} \frac{1}{\sqrt{2\pi}\sigma} \sum_{A}^{i} \Delta E_i R_i e^{[-(E-\Delta E_i)^2/(2\sigma)^2]}$$

Cell culture and differentiation

3T3-L1 preadipocytes were purchased from the American Type Culture Collection (ATCC® CL-173™) and cultured in growth medium supplemented with 10% bovine calf serum (BCS) and 1% penicillin/streptomycin (P/S) in Dulbecco's modified Eagle's medium (DMEM). At 70–80% confluency, which was designated as day 0, the 3T3-L1 cells were incubated in DMEM supplemented with 10% fetal bovine serum (FBS), 1% P/S, 0.5 mM 3-isobuyl-1-methylxanthine (IBMX), 1 µM dexamethasone, and 10 µg/mL insulin to allow their differentiation into mature adipocytes. Two days after the initiation of culture, the medium was replaced on alternate days with DMEM supplemented with 10% FBS, 1% P/S, and 10 µg/mL insulin. Throughout the process of adipogenesis, the cells were treated with compound **1** to evaluate their effects on adipogenesis.
